# Glucagon‐like pepetide‐1 receptor agonist suggests novel therapeutic options for hypothalamic obesity

**DOI:** 10.1111/jdi.14372

**Published:** 2024-12-11

**Authors:** Wanlu Ma, Bo Zhang, Xiaoping Chen

**Affiliations:** ^1^ Department of Endocrinology China‐Japan Friendship Hospital Beijing China

## Abstract

We briefly summarizes the mechanism of GLP‐1RA therapy in HO both in rodents and in humans. We also summarized the clinical trials and case reports of GLP‐1RA therapy in HO, especially the more and more often used semaglutide. We are hoping the therapy of GLP‐1RA in HO will arouse more attention from clinicians in the future.

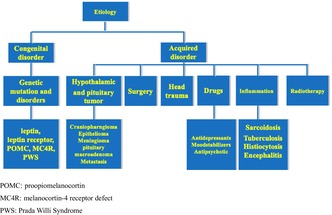

The incidence rate of obesity has increased year by year in recent years. People with obesity are more likely to develop diabetes and metabolic syndrome. Obesity combined with diabetes will have a higher risk of cardiovascular disease. The hypothalamus plays a pivotal role in regulating food intake, body weight, and energy expenditure. As the central regulator of food intake and appetite, the hypothalamus receives signals from the gastrointestinal tract, liver, adipose tissue, and pancreatic islet cells, and also regulates appetite‐related areas in the brain. Damage of the hypothalamus will lead to progressive weight gain, which is termed hypothalamic obesity (HO). HO is a severe form of obesity characterized by fatigue, hyperphagia, decreased physical activity, decreased satiety, and metabolic disorder caused by dysfunction of the hypothalamus. The mechanisms of HO are complex including hyperphagia and defects in satiety that are regulated by hypothalamic areas such as ventromedial hypothalamus (VMN), paraventricular nuclei (PVN), arcuate nucleus (ARC), and the lateral hypothalamic area^1^. Severe obesity risk factors, such as large tumors and invasive resection, often damage the ARC, VMN, PVN, and dorsomedial nucleus (DMN), leading to weight gain, insulin resistance, and hyperphagia, which may be caused by central leptin resistance, decrease of sympathetic activity and metabolic rate, low energy expenditure, temperature dysregulation, and increased energy storage in adipose tissue.

HO is a relatively rare disorder with complex etiologies, primarily due to dysfunctions in the regulation of energy intake and expenditure, the autonomic nervous system, and peripheral hormonal signaling pathways. HO could be due to congenital and acquired disorders (Figure 1). Congenital disorder of HO mainly includes abnormalities in the leptin‐melanocortin pathway, leading to monogenic obesity syndrome, such as Prada–Willi syndrome (PWS), melanocortin‐4 receptor (MC4R) defect, leptin or proopiomelanocortin (POMC) deficiency, and Bardet–Biedl syndrome. Acquired disorder includes hypothalamic and pituitary tumors, surgery, trauma, radiation therapy, inflammation, and antidepressant drugs. The most common causes are hypothalamic injury and tumors, such as craniopharyngioma, which may lead to hypopituitarism and hypothalamic nucleus damage. Even if pituitary dysfunction could be improved with hormone replacement therapy, 50% of patients still experience hyperphagia and severe obesity, which may be due to hypothalamic nucleus damage. Patients with craniopharyngioma have a 3–19 fold increased risk of cardiovascular death compared to normal individuals, and an increased risk of cerebral infarction, type 2 diabetes mellitus, and nonalcoholic fatty liver disease compared to the control group^2^. Patients with HO are more likely to have insulin resistance, abnormal glucose tolerance, diabetes, abnormal lipid levels, and increased risk of cardiovascular and cerebrovascular diseases. HO is more difficult to tackle than other forms of obesity as damage of hypothalamic nucleus leads to profoundly disturbed energy homoeostasis and severe loss of control over food intake. Early detection and effective treatment is of great importance.

**Figure 1:**
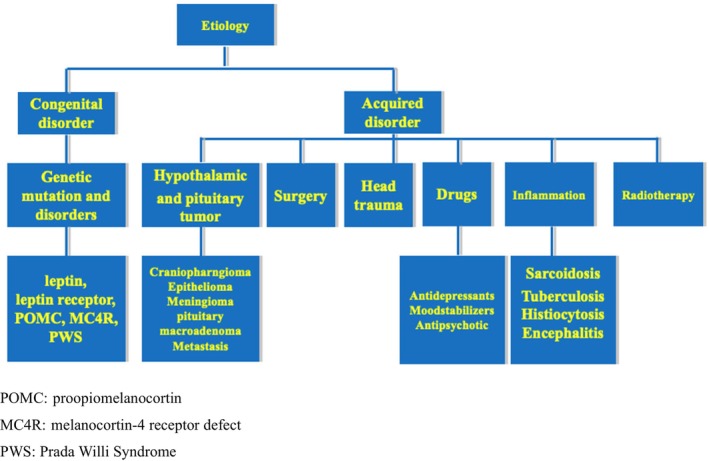
The etiology of hypothalamic obesity. The etiology of hypothalamic obesity is divided into congenital and acquired disorders. Congenital disorder of HO is mainly due to genetic mutation and disorders, such as Prada–Willi syndrome (PWS), melanocortin‐4 receptor (MC4R) defect, leptin or proopiomelanocortin (POMC) deficiency, and Bardet–Biedl syndrome. Acquired disorder includes hypothalamic and pituitary tumors, surgery, head trauma, radiation therapy, inflammation, and antidepressant drugs. Hypothalamic and pituitary tumors include craniopharngioma, epithelioma, meningioma, pituitary macroadenoma, and metastasis. Hypothalamic and pituitary inflammation includes sarcoidosis, tuberculosis, histiocytosis and encephalitis. Antidepressant drugs include antidepressants, moodstabilizers and antipsychotic.

From the discovery of an intestinal extract that could produce hypoglycemia in 1930, to the flourishing development of different kinds of Glucagon‐like pepetide‐1 receptor agonist (GLP‐1RA) advocated in diabetes in recent years, GLP‐1RA has undergone a long way in the application of diabetes. GLP‐1RA can act on different tissues and organs throughout the body, therefore exerting multiple effects. It can promote insulin secretion by effecting on pancreatic β cells and reduce secretion of glucagon in a glucose dependent manner, lower body weight, lower blood pressure, and blood lipids, and protect the myocardium. Nowadays, GLP‐1RA has been used more and more for people with type 2 diabetes mellitus and obesity.

The application of GLP‐1RA in the treatment of HO is a rather novel approach. GLP‐1RA functions well on HO because it does not depend on intact hypothalamic structure to effect. GLP‐1 plays the role of satiety hormone, reduces food intake, promotes rapid meal termination, and modulates activity in appetite and reward‐related brain areas including hypothalamus, hindbrain, hippocampus, and mesolimbic brain reward system by binding to receptors in the vagus nerve and appetite‐related sites in the hindbrain, ARC and DMN of hypothalamus^3^. Also, GLP‐1RA may activate catecholamine neurons in postrema and change the sympathetic/parasympathetic tone balance. GLP‐1R is mostly distributed in the PVN and ARC of mice, while brain‐derived GLP‐1 exerts anorexigenic and satiety effects in PVN^4^. Injection of GLP‐1 (7–36) into PVN suppressed feeding in mice^5^. After intravenous injection of semaglutide in C57BL/6 mice, clear signals were detected in brain regions related to reward behavior and food intake. After subcutaneous injection of semaglutide in obese mice, neurons related to food intake and reward behavior were significantly activated compared to the control group, indicating that semaglutide acts on the hypothalamus and exerts appetite suppressing effects. Semaglutide modulates food preference, reduces food intake, and causes weight loss but not on energy expenditure^6^.

The expression of GLP‐1R in the human body varies in different people. GLP‐1R expression in the lateral hypothalamic nucleus decreases in patients with body mass index (BMI) more than 25 kg/m^2^, and GLP‐1R expression in PVN and ARC decreases in patients with type 2 diabetes mellitus^7^. After intravenous injection of exenatide in obese (with normal blood glucose) and patients with type 2 diabetes mellitus, the response to food in the brain's insula, amygdala, putamen, and orbitofrontal cortex (involved in feeding and reward behavior) regions decreases^8^.

Clinical studies on the application of GLP‐1RA on HO are limited. The first study recruited nine patients with moderate to severe HO, in which eight cases were complicated with type 2 diabetes mellitus. The nine patients were treated with exenatide (eight) or with liraglutide (one) for 51 months. Eight patients experienced substantial weight loss, improved insulin resistance, and decreased HbA1c, while five patients experienced increase of satiety after treatment^9^. Case reports also indicate that after application of exenatide (2 mg per week) in children with HO, one patient reached weight loss of 5.4 kg and two patients experienced improvement of hyperorexia. One case report indicated the BMI of an adult patient with HO decreased by 6.5 kg/m^2^ after 8‐month liraglutide treatment. The Energy Balance and Weight Loss in Craniopharyngioma‐related or Other Hypothalamic Tumors in Hypothalamic Obesity (ECHO) trial was later on launched to further elucidate the discovery. The randomized, dual arm, multicenter, double‐blind, placebo‐controlled ECHO trial was conducted on 42 patients aged 10–25 years with hypothalamic injury and HO caused by intracranial tumors^10^. A decrease in body fat mass and waist circumference was observed in the treatment group (exenatide 2 mg once a week) with no significant difference in BMI between two groups. The trial further found that energy intake and total energy expenditure decreased after 36 weeks of treatment with exenatide for HO^11^. This study further evaluated the impact of hypothalamic injury severity on the efficacy of exenatide treatment. A more significant decrease in fat content after exenatide treatment could be observed in patients with poorer hypothalamic injury score (HLS score), more severe nipple damage, and higher basal fat content^12^. A multicenter, 52‐week, placebo‐controlled trial recruited 31 adolescents aged 12–17 years and 24 children aged 6–11 years with PWS and obesity. And hyperphagia total and drive scores were lower in adolescents treated with liraglutide compared to no treatment^13^. With semaglutide more and more often used in obese and diabetes patients, certain case reports also observed the application of semaglutide in patients with HO. A 16‐year‐old male patient of craniopharyngioma who underwent trans‐sphenoid sinus surgery gained sufficient weight loss (123 → 92 kg), decreased appetite, and attenuated loss of control before food after the treatment of semaglutide 2 mg once a week for 6 months^14^. Another case is a 45‐year‐old female diagnosed with enamel craniopharyngioma. She also gained a weight loss of 10 kg (96 → 86 kg) after 12 months of treatment of semaglutide 2 mg once a week^15^.

HO is a complex and challenging clinical condition that requires further research and attention. With new drugs of obesity and diabetes evolving quickly in recent years, more interventions and drugs are forming the landscape of HO. The GLP‐1 and GIP receptor agonists, along with the triple agonist of GIP, GLP‐1, and glucagon receptor, showed good efficacy in promoting weight loss in obese patients with type 2 diabetes mellitus. New drugs based on GLP‐1 and bariatric surgery are expected to play more and more important roles in HO treatment in the future.

## DISCLOSURE

The authors declare no conflict of interest. Chen Xiaoping is an Editorial Board member of JOURNAL OF DIABETES INVESTIGATION and a co‐author of this article. To minimize bias, they were excluded from all editorial decision‐making related to the acceptance of this article for publication.

Approval of the research protocol: N/A.

Informed consent: N/A.

Registry and the registration no. of the study/trial: N/A.

Animal studies: N/A.

## FUNDING

National High Level Hospital Clinical Research Funding and Elite Medical Professionals Project of China‐Japan Friendship Hospital (No. ZRJY2021‐QM02); National High Level Hospital Clinical Research Funding (2022‐NHLHCRF‐LX‐02‐0101); Young Elite Scientist Sponsorship Program By Bast (No. BYESS2023173).
